# Dynamic-budget superpixel active learning for semantic segmentation

**DOI:** 10.3389/frai.2024.1498956

**Published:** 2025-01-09

**Authors:** Yuemin Wang, Ian Stavness

**Affiliations:** Department of Computer Science, University of Saskatchewan, Saskatoon, SK, Canada

**Keywords:** dynamic-budget querying, superpixel, regional querying, active learning, semantic segmentation

## Abstract

**Introduction:**

Active learning can significantly decrease the labeling cost of deep learning workflows by prioritizing the limited labeling budget to high-impact data points that have the highest positive impact on model accuracy. Active learning is especially useful for semantic segmentation tasks where we can selectively label only a few high-impact regions within these high-impact images. Most established regional active learning algorithms deploy a static-budget querying strategy where a fixed percentage of regions are queried in each image. A static budget could result in over- or under-labeling images as the number of high-impact regions in each image can vary.

**Methods:**

In this paper, we present a novel dynamic-budget superpixel querying strategy that can query the optimal numbers of high-uncertainty superpixels in an image to improve the querying efficiency of regional active learning algorithms designed for semantic segmentation.

**Results:**

For two distinct datasets, we show that by allowing a dynamic budget for each image, the active learning algorithm is more effective compared to static-budget querying at the same low total labeling budget. We investigate both low- and high-budget scenarios and the impact of superpixel size on our dynamic active learning scheme. In a low-budget scenario, our dynamic-budget querying outperforms static-budget querying by 5.6% mIoU on a specialized agriculture field image dataset and 2.4% mIoU on Cityscapes.

**Discussion:**

The presented dynamic-budget querying strategy is simple, effective, and can be easily adapted to other regional active learning algorithms to further improve the data efficiency of semantic segmentation tasks.

## 1 Introduction

Advances in deep learning have steadily improved performance on general computer vision datasets like Cityscapes (Cordts et al., 2016). Adapting this success to a more specialized area like precision agriculture usually requires creating a sizable and well-labeled dataset. Abundant images can be relatively cheaply collected while manually labeling thousands of images can be costly, tedious and prone to human errors caused by repetition and boredom. Furthermore, the benefit of additional labeled data often exhibits diminishing returns when the dataset grows unnecessarily big (Sun et al., 2017) due to easy and low-impact images/regions being labeled. This diminishing return is particularly problematic in agricultural datasets where class imbalance is common. Only 757 images out of the 12,330 images in the SugarBeets 2,016 dataset (Chebrolu et al., 2017) contain weeds making the rest of the labeled images less beneficial for model training.

The application of deep learning also faces challenges in generalizability due to complex background/soil conditions, differences in vegetation species and volunteer weeds. Models trained on one farm field often fail on other distinct fields. We often have to repeat the entire pipeline of data curation, labeling and model training for each field leading to increased labeling and computational costs. Deep learning data efficiency can be greatly improved by prioritizing high-impact images/regions images that have the highest positive impact on model accuracy for labeling. Active learning (AL) (Cohn et al., 1994) is a well-known technique for selectively labeling high-impact data points. This allows us to effectively train models with significantly smaller datasets, and simultaneously decrease labeling and computational costs.

Many active learning algorithms have been proposed for image classification (Gal et al., 2017; Krishnamurthy et al., 2019; Sener and Savarese, 2017b; Vijayanarasimhan and Grauman, 2009) and have inspired dedicated AL algorithms for semantic segmentation. Semantic segmentation is crucial for agricultural field images due to dense, tangled and occluded plants; also important for precision agriculture where crop and weed plants need to be differentiated, labeling is very costly because it often requires plant science expertise to differentiate between crop/weed instances with similar appearance.

The AL algorithms designed for semantic segmentation typically take advantage of a pixel-level uncertainty map (Mackowiak et al., 2018) to perform regional querying on images. Querying is a process in active learning algorithms where the learner (model) determines and selects high-impact data points to be labeled by the oracle (human labeller). Regional querying only queries high-impact regions from high-impact images, partially labeling images and further lowering the labeling cost.

Instead of querying rectangular regions like Mackowiak et al. (2018), we can also query superpixels. Cai et al. (2021) compared different regional querying algorithms and concluded that superpixel querying is more cost-effective. Siddiqui et al. (2020) proposed an effective superpixel querying AL algorithm which ranks all of the regions in the unlabelled pool. This can incur a significant overhead since with *n* images and *m* regions per image (in the thousands for high-resolution images) the sorting time complexity is *nmlog*(*nm*). We opt to rank whole images first and then from the selected *b* images, rank the regions within these images. The overhead of our method is reduced to *nlog*(*n*)+*bmlog*(*m*), where *b* is a small number (50 in our low-budget experiment). Both Mackowiak et al. (2018) and Cai et al. (2021) inspired our proposed algorithm where we extended the querying process of the superpixel-based active learning algorithm to use a dynamic per-image budget.

Much of the prior work in active learning, including the studies mentioned above, evaluates their AL approaches within limited-scope experiments, e.g. with a single dataset, or a single budget range, or constant region size. Furthermore, the effect of querying region sizes is largely overlooked. We addressed these gaps by experimenting with low- and high-budget scenarios, as well as testing with both coarse and fine superpixels for both datasets.

In this paper, we introduce a novel dynamic-budget superpixel querying strategy to improve regional querying active learning algorithms. This strategy uses clustering to dynamically query the optimal amount of superpixels from the queried images. We demonstrate the effectiveness of this strategy by evaluating both low- and high-budget scenarios with two distinct datasets: a general dataset of street scenes (Cityscapes) and a more specialized dataset of agriculture field images (Nassar 2020) (Wang et al., 2023). The source code of this project is publicly available at: https://github.com/yuw422/dynamic-budget-superpixel-active-learning.git.

The main contributions of our paper include:

- We proposed a novel dynamic-budget querying strategy that can improve regional active learning algorithms for semantic segmentation.
- We evaluated our querying strategy on a specialized agricultural field image dataset (Nassar) and a general street scene dataset (Cityscapes).
- We evaluated our querying strategy in both low- and high-budget scenarios.
- We demonstrated the effects of superpixel sizes on our querying strategy.

## 2 Materials and methods

In this section we will introduce the overall active learning algorithm, the proposed querying strategy and the foreground-only querying strategy designed for the specialized dataset. We also describe the two datasets (Nassar 2020 and Cityscapes) and the two models (UNet and DeepLabv3+) used in this paper. Lastly, we explain the different experiment scenarios.

### 2.1 Datasets

We evaluate our proposed algorithm on two distinct semantic segmentation datasets: Nassar 2020 (Wang et al., 2023), a specialized agricultural field image weed detection dataset and Cityscapes (Cordts et al., 2016), a popular general street scenes dataset. Cityscapes is a considerably more complex dataset compared to Nassar 2020, with higher definition images and more classes. Most Cityscapes images are crowded with objects whereas Nassar images are very background-dominant. The significant differences between these two datasets can provide us with a better understanding of how our algorithm performs with different types of data.

#### 2.1.1 Nassar 2020

The Nassar 2020 dataset is a UAV-collected weed detection dataset collected from an experimental wheat field. This dataset is composed of six high-definition images of various sizes sampled from the whole-field orthormosaic image. Each image in this dataset comes with pixel-level labels in one of the three classes: background (soil), crop, and weed.

For ease of use, we chose a tiled version of this dataset where each image in the dataset is cut into 256 × 256 tiles with no tile overlaps. We use the pre-determined data split of this tiled dataset with 1,784 images for training, 168 images for validation and 266 images for testing. One interesting aspect of the Nassar dataset is that the three classes of interest can be divided into two categories: background (mainly soil) and foreground which contains all vegetation pixels including crops and weeds.

**Figure 1:**
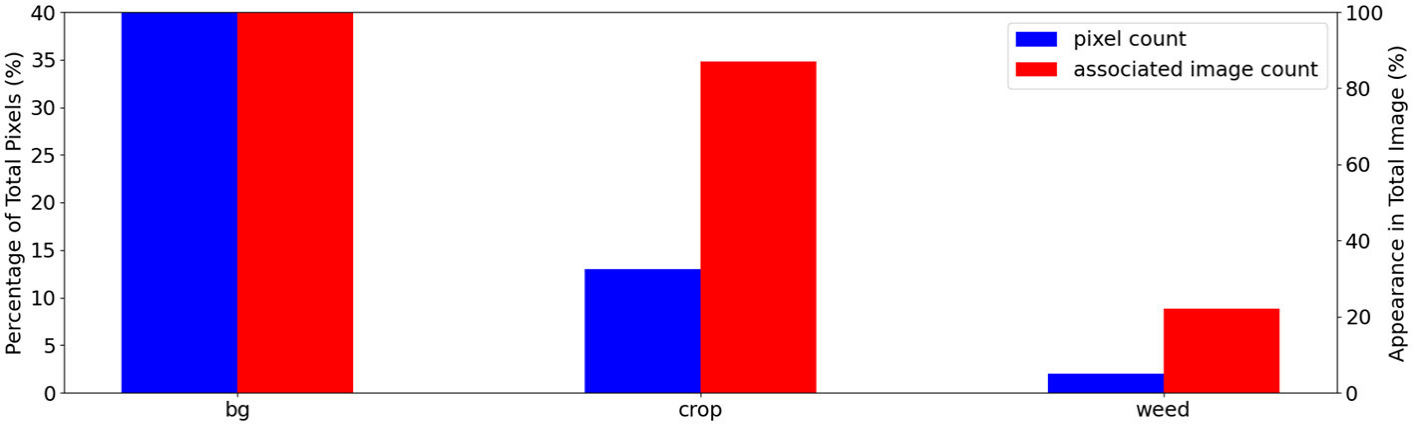
Nassar class distribution plot showing the class imbalance in this dataset: total class pixel count in blue and associated image count in the training set images in red. We show the pixel count and associated image count as a percentage of the total pixel/image count. A static-budget querying scheme could over-labeling the abundant crop class while under-labeling the under-represented weed class.

**Figure 2:**
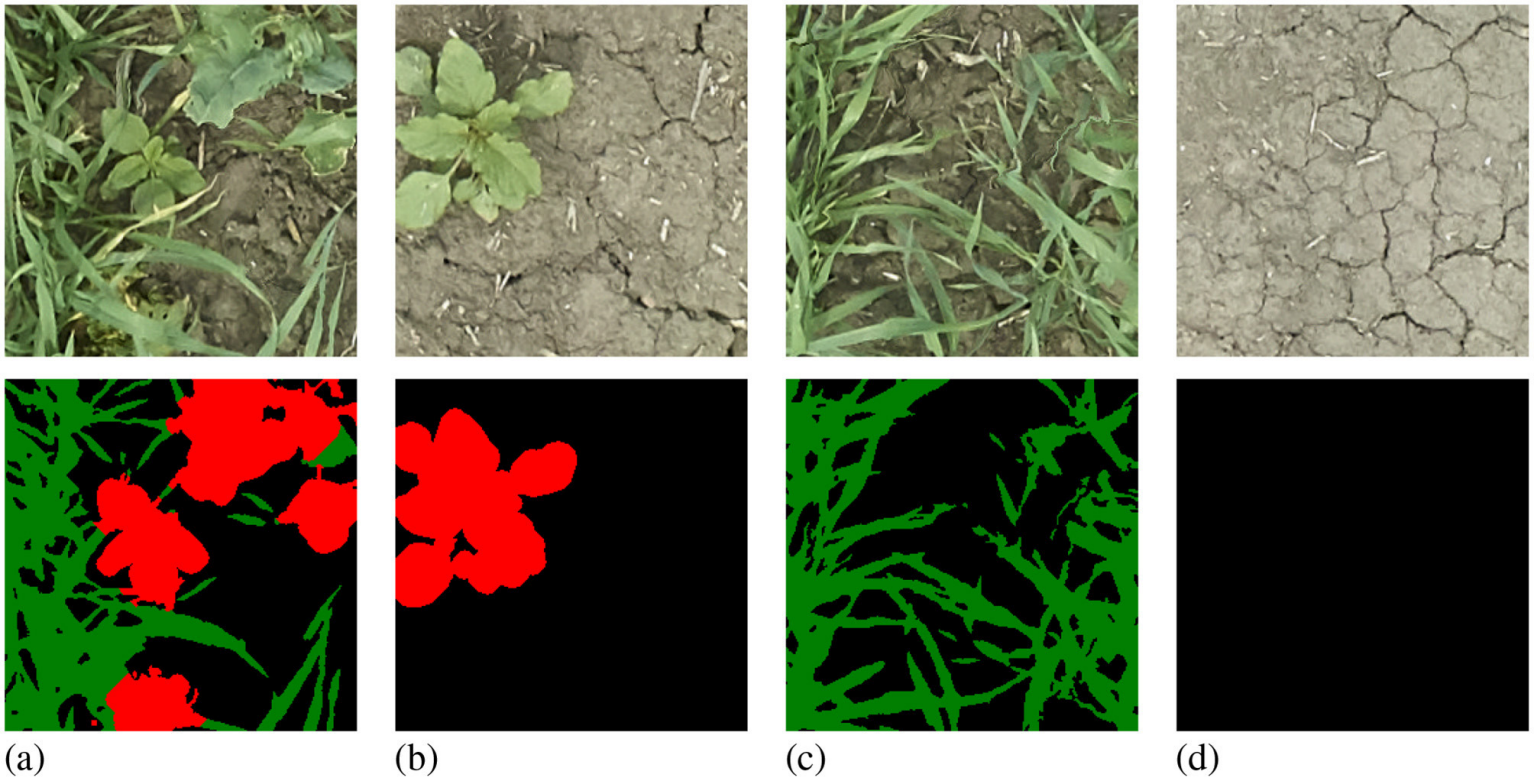
Samples from the Nassar 2020 dataset with labels: background in black, crop in green, and weed in red. The chosen examples show a sample with balanced crop and weed pixels **(A)**, a sample with no crop pixels **(B)**, a sample with no weed pixels **(C)**, and a sample with only background pixels **(D)**.

The dataset is highly background-dominant as shown in Figure 1, where background pixels make up 85% of all pixels in the training set and appear in every single image. The foreground pixels are also imbalanced: in the training set, 13% are crop pixels that appear in 87% of images compared to only 2% of weed pixels found in 22% of images. Figure 2 shows examples of images in the Nassar dataset that contain no crop or weed pixels making the dynamic-querying strategy less effective as mentioned in Section 2.2.3.

#### 2.1.2 Cityscapes

The Cityscapes dataset contains a large amount of street scene images collected from 50 different cities. There are several collections in this dataset covering different camera positions and image definitions. We selected the 8-bit collection from the left-side camera with fine pixel-level annotations.

This dataset comes with a pre-determined split of 2,975 training images, 500 validation images and 1,525 testing images of size 2,048 × 1,024. Since the testing images do not come with labels, we use the 2,975 training images as our training and validation sets and the 500 validation images as the test set. The Cityscapes dataset contains a total of 35 labeled classes with an ignore class. The author of Cityscapes picked 19 out of the 35 labeled as classes of interest and we used the provided class map to convert the provided 35-class labels into the desired 19-class training labels. We chose to downsample the Cityscape images by half (1,024 × 512) by uniformly dropping pixels to save time and GPU memory and cast any labels that are not of the 19 classes of interest to the ignore class. The downsampling in theory can affect the absolute performance of our model but we are only interested in the relative performance difference between the two discussed querying schemes. Since all the experiments use the same downsampled dataset, the downsampling should not affect our results.

**Figure 3:**
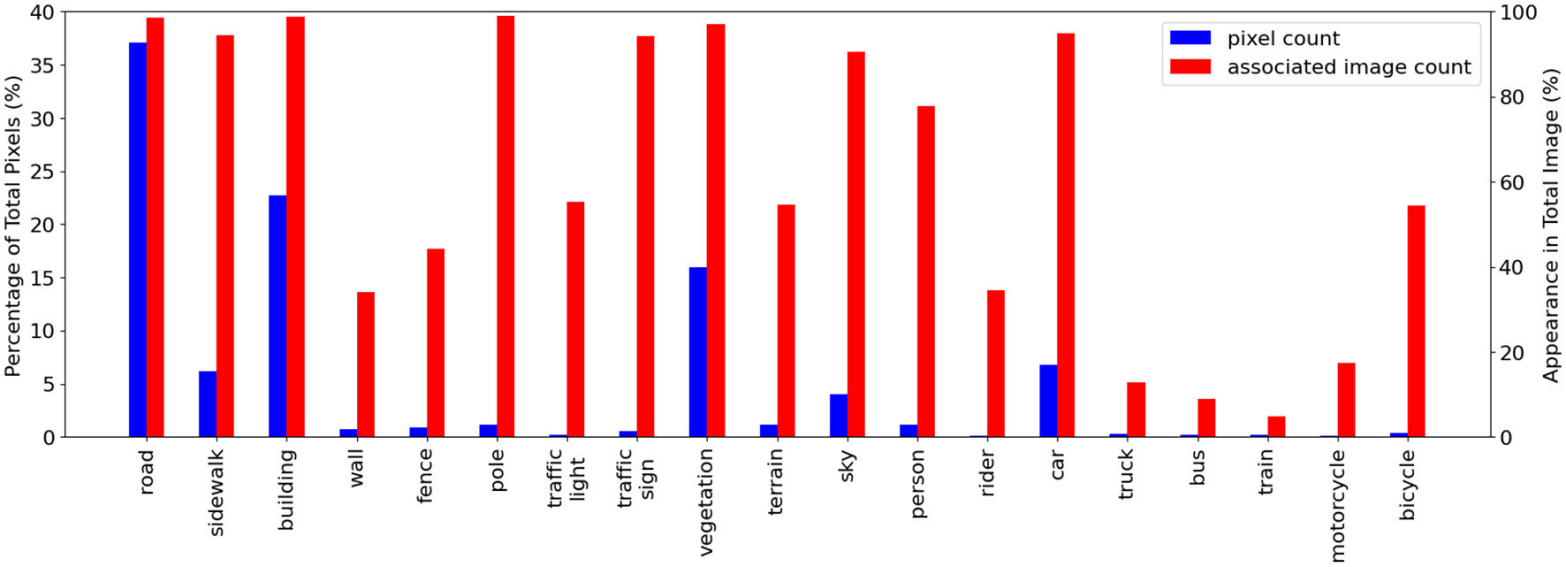
Cityscapes class distribution plot showing the class imbalance in this dataset: total class pixel count in blue and associated image count in the training set images in red. We show the pixel count and associated image count as a percentage of the total pixel/image count. Dynamic-budget querying could help to avoid over-labeling dominant classes like road and building. Classes like the fence with medium image appearance frequency but low pixel count could benefit from a dynamic querying budget where additional labeling could more effectively capture all the fence pixels.

Cityscapes is also highly imbalanced between its 19 classes both in pixel counts and image appearance frequencies as shown in Figure 3. The common road pixels make up around 37% of total pixels and appear in 99% of training images. Other classes appear considerably less. Motorcycle pixels make up 0.09% of total pixels and appear in 17.3% training images while bus pixels only appear in 9% of training images and contribute to 0.22% of total pixels.

This class imbalance indicates each image is likely to have a different amount of high-impact regions, and the less common classes are usually more desirable for labeling. Enforcing a fixed querying budget for each image could result in under-labeling, where the image has more high-impact regions than the budget resulting in the discarding of some high-impact regions, or over-labeling, where the image has fewer high-impact regions than the budget resulting in the inclusion of low-impact regions. For example, if our dataset already contains many images with cars and the AL is presented with an image with one bicycle and ten cars. Ideally, we only want to label the bicycle as the dataset already contains many car examples. Compared to the Nassar dataset where it is obvious that the under-represented weed class is more desirable for labeling, the more complex Cityscapes dataset has a mix of high frequency, high pixel count classes; high/medium frequency, low pixel count classes and low frequency and low pixel count classes. Using a dynamic querying budget allows the AL process to more effectively capture the low pixel counts classes like the fence and the truck, regardless of their appearance frequencies. It makes intuitive sense to use a dynamic per-image querying budget to label each image optimally.

### 2.2 Dynamic-budget querying active learning

Since our study focuses on dynamic-budget querying in AL, we isolate the effect of dynamic vs static budgeting by using a standard uncertainty sampling AL algorithm for each condition. The overall active learning algorithm is composed of an initialization step and several active learning steps. For our active learning experiments, the initialization training follows the same procedures from the full dataset baselines: UNet is trained from scratch with no augmentation while DeepLabv3+ is pre-trained and uses augmentations. In each active learning step, instead of training the model from the initialization state, we use the trained weights from the previous active learning step as pretraining. It should be pointed out that we do not use human labellers in our AL experiments, but instead simply make the already available labels visible to our model to simulate the labeling process. This is commonly done in AL experiments to both reduce cost and ensure label quality.

#### 2.2.1 Initialization step

We start with an unlabelled pool containing all of the unlabelled images and an empty AL training set. To initialize the algorithm, we randomly select a small batch of images from the unlabelled pool, give these selected images whole-image manual labels, and add them to our AL training set. These initialization images are the only images to receive whole-image labels throughout the entire active learning process. We train our model on this initialization training set until convergence to finish the initialization step.

#### 2.2.2 Active learning step

Once the initialization step is complete, we repeat active learning steps until a predetermined budget is met. The active learning step contains an image querying step followed by a superpixel querying step. In each active learning step, we want to find the highest-impact superpixels in the highest-impact images from our unlabeled pool. The superpixel and image impactfulness is measured with uncertainty using the acquisition function Bayesian Active Learning by Disagreement (BALD), whereas higher impactfulness has higher uncertainty. The BALD acquisition function takes T samples using MC-Dropout and then combines the mean of the sample-wise pixel entropy and the entropy of the mean of the sample pixels. The image and superpixel uncertainties are aggregated using their member pixels' uncertainties.

In each AL step, with an unlabeled pool containing *n* images and *m* regions per image, we want to find *b* images with the highest uncertainty, and for each image *q* most uncertain superpixels. After the MC sampling, each pixel in an image contains samples of class distributions $P_{t,c}^{\left(u,v\right)}$ at pixel (*u, v*) with *T* samples and *C* classes. The pixel-wise Bayesian Active Learning by Disagreement (BALD) uncertainty *U*^(*u, v*)^ is calculated as


(1) $$\begin{array}{l} U^{\left(u,v\right)}=\frac{1}{T}\overset{T}{\underset{t=1}{\sum }}E\left(P_{t,c}^{\left(u,v\right)}\right)+E\left(\frac{1}{T}\overset{T}{\underset{t=1}{\sum }}P_{t,c}^{\left(u,v\right)}\right) \end{array}$$

where *E*(*P*_*c*_) is the entropy of class probabilities *P*_*c*_ defined as


(2) $$\begin{array}{l} E\left(P_{c}\right)=-\underset{c}{\sum }P_{c}log\left(P_{c}\right) \end{array}$$

Then the image uncertainty *U*^(*i*)^ can be aggregated as


(3) $$\begin{array}{l} U^{\left(i\right)}=\frac{1}{U}\frac{1}{V}\underset{u}{\sum }\underset{v}{\sum }U^{\left(u,v\right)} \end{array}$$

where U and V are the horizontal and vertical pixel counts. Similarly, we aggregate the superpixel uncertainty *U*^(*s*)^ as the mean of its member pixels' uncertainty. We use the Jenks natural breaks (Jenks, 1967) algorithm to split the superpixels into high- and low-uncertainty clusters because the pixel uncertainty visualizations intuitively show bright high-uncertainty pixels and dark low-uncertainty pixels. We briefly tried three clusters per image aiming to further reduce labeling costs but this harsher threshold resulted in under-labeling.

**Figure 4:**
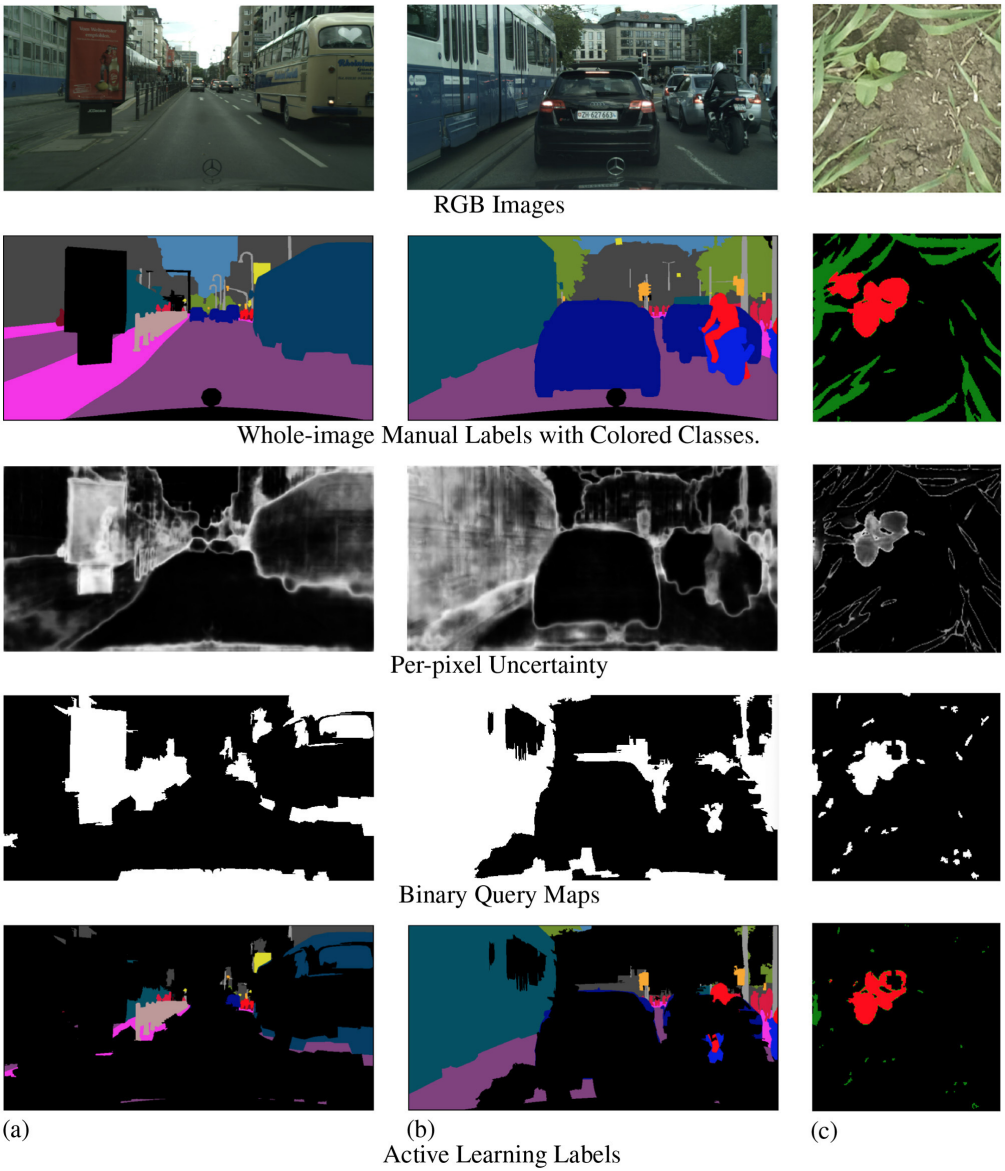
Examples from Cityscapes **(A, B)** and Nassar **(C)** showing the querying process in active learning steps. The first row is RGB images and the second row is whole-image manual labels. The third row shows the per-pixel uncertainty for each image scale to [0, 1], with black showing 0 and white showing 1. The fourth row contains the query maps where high-uncertainty superpixels are in white and low-uncertainty superpixels are in black. The last row is the resulting active learning labels where the high-uncertainty superpixels from the query masks are given manual labels while the rest of the image is ignored.

Once we obtain the high- and low-uncertainty superpixel clusters, we query and then manually label all the high-uncertainty superpixels in the image. The remaining low-uncertainty superpixels are automatically labeled to the ignore class. We then add this batch of partially manually labeled images to our AL training set and use this updated AL training set to train our model until convergence. At the end of each active learning step, we test the trained model on the test set to record AL step mIoU. Each stage of the AL step is visualized in Figure 4.

#### 2.2.3 Foreground-only querying for specialized dataset

For our specialized weed detection dataset, we made a few improvements to the algorithm to adapt to the dataset's characteristics. As explained in Section 2.1.1, the Nassar dataset is very background-dominant. The background soil pixels can be easily separated from the foreground vegetation pixels by applying thresholding to a vegetation index like Color Index of Vegetation (CIVE) (Kataoka et al., 2003). Because of the color difference between foreground and background pixels, our model can achieve near-perfect accuracy on the background class right after the initialization training. This also means all the background pixels will have extremely low uncertainty scores.

In practice, directly applying the active learning step described in Section 2.2.2 will result in the natural breaks algorithm simply clustering the background pixels into the low-uncertainty cluster and the foreground vegetation pixels into the high-uncertainty cluster. This means we are querying almost all foreground vegetation pixels which makes the algorithm ineffective since the difficulty of labeling the Nassar dataset is separating the foreground pixels into crop and weed. We modified our algorithm to use the model's background prediction to exclude background pixels in both image ranking and superpixel ranking. We take a further step to scale the per-image uncertainty score with the image's foreground pixel percentage. This scaling forces our image querying to balance between superpixel uncertainty and foreground pixel presence.

### 2.3 Models

In this project, we chose two popular semantic segmentation models: UNet (Ronneberger et al., 2015), and DeepLabv3+ (Chen et al., 2018). The UNet model is used on the specialized weed detection Nassar 2020 dataset while the more capable DeepLabv3+ model with Xecption backbone is chosen for Cityscapes. We chose the less complex UNet model for the simpler Nassar dataset since it can be trained efficiently but still yield high accuracy. However, the UNet model struggles with the considerably more difficult Cityscapes dataset. We are not confident with a low accuracy baseline due to its randomness and chose the more capable DeepLabv3+ for Cityscapes to achieve a baseline close to the state-of-the-art benchmark. A low accuracy baseline contains more noise, making the baseline less stable and usually resulting in larger accuracy fluctuation between runs with the same setup. This makes it harder to determine whether the performance difference is caused by algorithmic changes or model instability. We train our models on the entire labeled dataset until converge to get our full dataset baseline.

Since the Nassar dataset is relatively easy to learn, we train our UNet model from scratch with no data augmentation. To give a more realistic evaluation of the more difficult Cityscapes dataset, we chose to use a PASCAL VOC pre-trained model and incorporate a list of simple augmentations: horizontal flip, random crop, random brightness, and random gamma. The pre-training and augmentation allow us to conduct our experiments as close to the state-of-the-art Cityscapes benchmarks as possible without having to deploy complicated training routines or specialized models. Both models are implemented (Hiasa et al., 2019; Yu et al., 2020) with MC-Dropout (Gal and Ghahramani, 2015, 2016) to approximate a Bayesian Neural Network (BNN) for better uncertainty estimation. MC-Dropout is a popular choice to estimate model uncertainty due to its simplicity (Mackowiak et al., 2018; Siddiqui et al., 2020) and reasonable effectiveness (Seoh, 2020). We evaluate the performance of our models using Mean Intersection over Union (mIoU).

In the Nassar experiments, we use a batch size of 4, a dropout rate of 0.5, and an initial learning rate of 1e-4. In the Cityscapes experiments, we use a batch size of 5, a dropout rate of 0.5, and an initial learning rate of 1e-4. All experiments are set up to train for a maximum of 500 epochs with an autostop where the val loss does not decrease for 40 consecutive epochs.

### 2.4 Experiments

In this section, we describe the experiments being conducted. Below are descriptions of the different scenarios we consider. Several scenarios can be combined in a single experiment run. For example, a run can be dynamic-budget, low-budget and using fine superpixels. All experiments with the same budget and dataset use the same initialization step model to ensure consistency in the initialization step. For example, in the Nassar low-budget experiments, we train our model on 50 randomly selected images in the initialization step. Once this is complete, the same checkpoint is shared by all low-budget Nassar experiments.

#### 2.4.1 Dynamic-budget experiments

##### 2.4.1.1 Low-budget Experiments

In a low-budget experiment, we label fewer images for the initialization step and active learning steps. The Nassar dataset experiments will have 50 images fully labeled for the initialization step and each active learning step queries an additional 50 images. In our preliminary experiments, we found that a step size of 50 images gives a detailed enough IoU curve progression while keeping running time low. We run the experiment for six active learning steps which results in 350 images being labeled. The Cityscapes low-budget experiments use the same initialization and active learning step sizes. For the low-budget experiments, we also include a baseline trained on 350 random images with whole-image manual labels.

##### 2.4.1.2 High-budget experiments

The high-budget experiments operate in percentages of the unlabelled pool instead of image counts. The high-budget experiments for both datasets will use 10% of the unlabelled pool for both initialization and each active learning step. Again, this step size gives a balance between enough details on the IoU progression and running time. This means the Nassar experiments will label 194 images for each step while the Cityscapes experiments label 296 images per step. We run the high-budget experiments for five active learning steps for Cityscapes and four active learning steps for Nassar. With 10% initialization data, the high-budget experiments stop when 70% of the unlabelled pool is labeled for Cityscapes and 50% for Nassar.

##### 2.4.1.3 Superpixel sizes experiments

We experiment with fine and coarse superpixels for both datasets. For the Nassar dataset, we test 2,000 (fine) and 500 (coarse) superpixels per image. The Cityscapes images are much larger than the Nassar images which allows us to segment each image into more superpixels. We test 5,000 (fine) and 500 (coarse) superpixels per image for the Cityscapes experiments. We will only compare fine and coarse superpixels with dynamic-budget querying since it outperforms static-budget querying and we are interested in whether using coarse superpixels will impact the performance.

#### 2.4.2 Static-budget experiments

We compare our dynamic-budget querying scheme with the typically used static-budget querying. The average percentage of labeled superpixels per image is used as a proxy for labeling cost measurements. We record the average percentage of labeled superpixels per image for the dynamic-budget querying experiment and use the same value in the static-budget querying experiment. Doing this keeps the number of superpixels labeled in the static- and dynamic-budget querying experiments very close. For the Nassar dataset, we only count the percentages of labeled foreground superpixels per image since the algorithm only operates on foreground pixels. We ran the static-budget experiments following the same procedure as described in Section 2.4.1.1 for the low-budget setting and Section 2.4.1.2 for the high-budget setting.

## 3 Results

**Figure 5:**
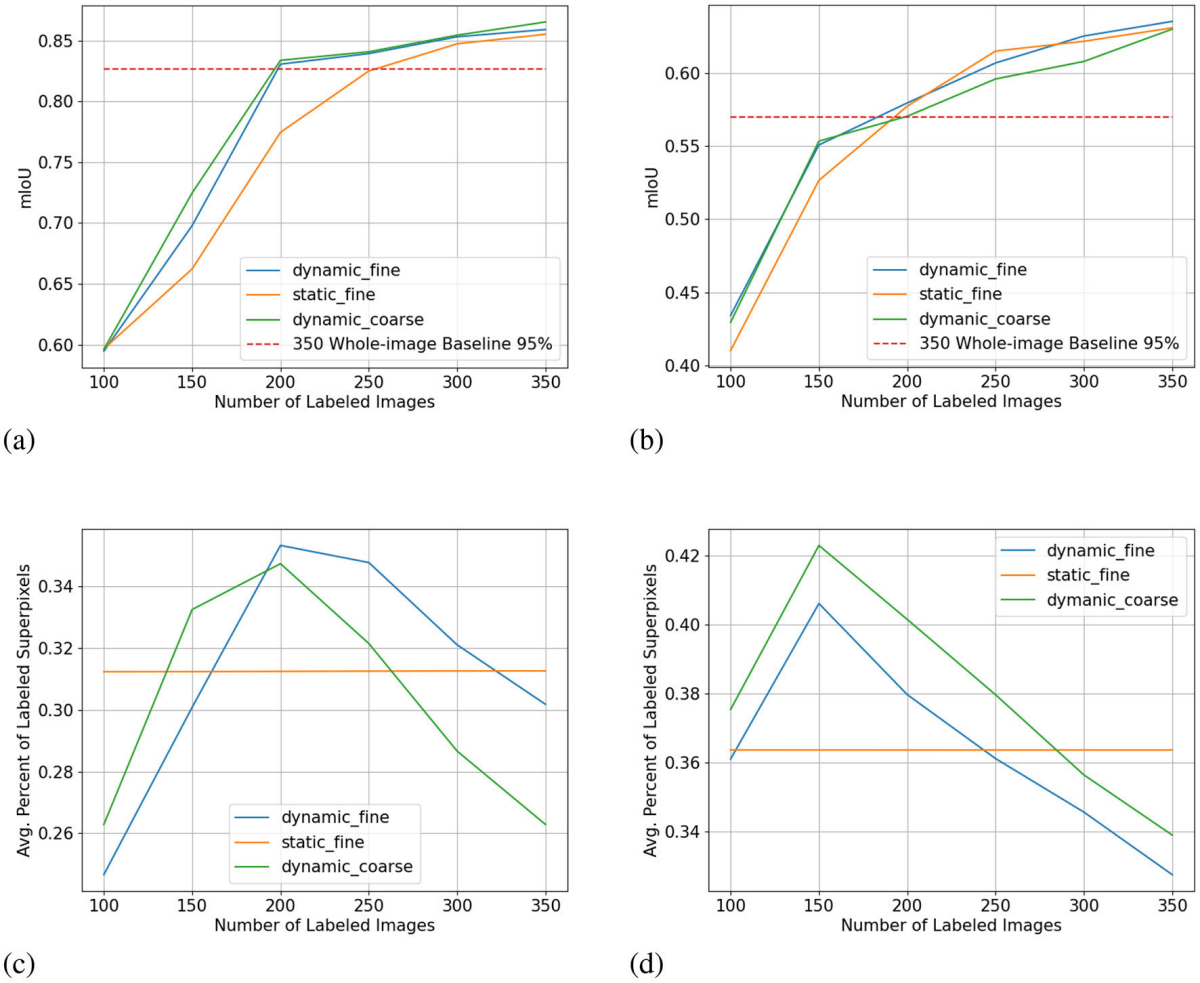
Low-budget experiment results showing mIoU (top row, higher is better) and average percentage of labeled superpixels (bottom row) for the Nasser (left) and Cityscapes (right) dataset. Label dynamic_fine refers to dynamic-budget experiments using fine superpixels; label dynamic_coarse refers to dynamic-budget experiments using coase superpixels; label static_fine refers to experiments using fine superpixels. X-axis for all subplots is the number of labeled images. The average percentage of labeled superpixels is calculated at the end of each active learning step as the number of labeled superpixels over the number of total superpixels. A lower average percentage of labeled superpixels is better as it means a lower budget. This represents on average, what percentage of superpixels in an image are manually labeled. **(A)** mIoU on Nassar 2020. **(B)** mIoU on Cityscapes. **(C)** Avg. % Labeled Superpixles on Nassar 2020. **(D)** Avg. % Labeled Superpixles on Cityscapes.

**Figure 6:**
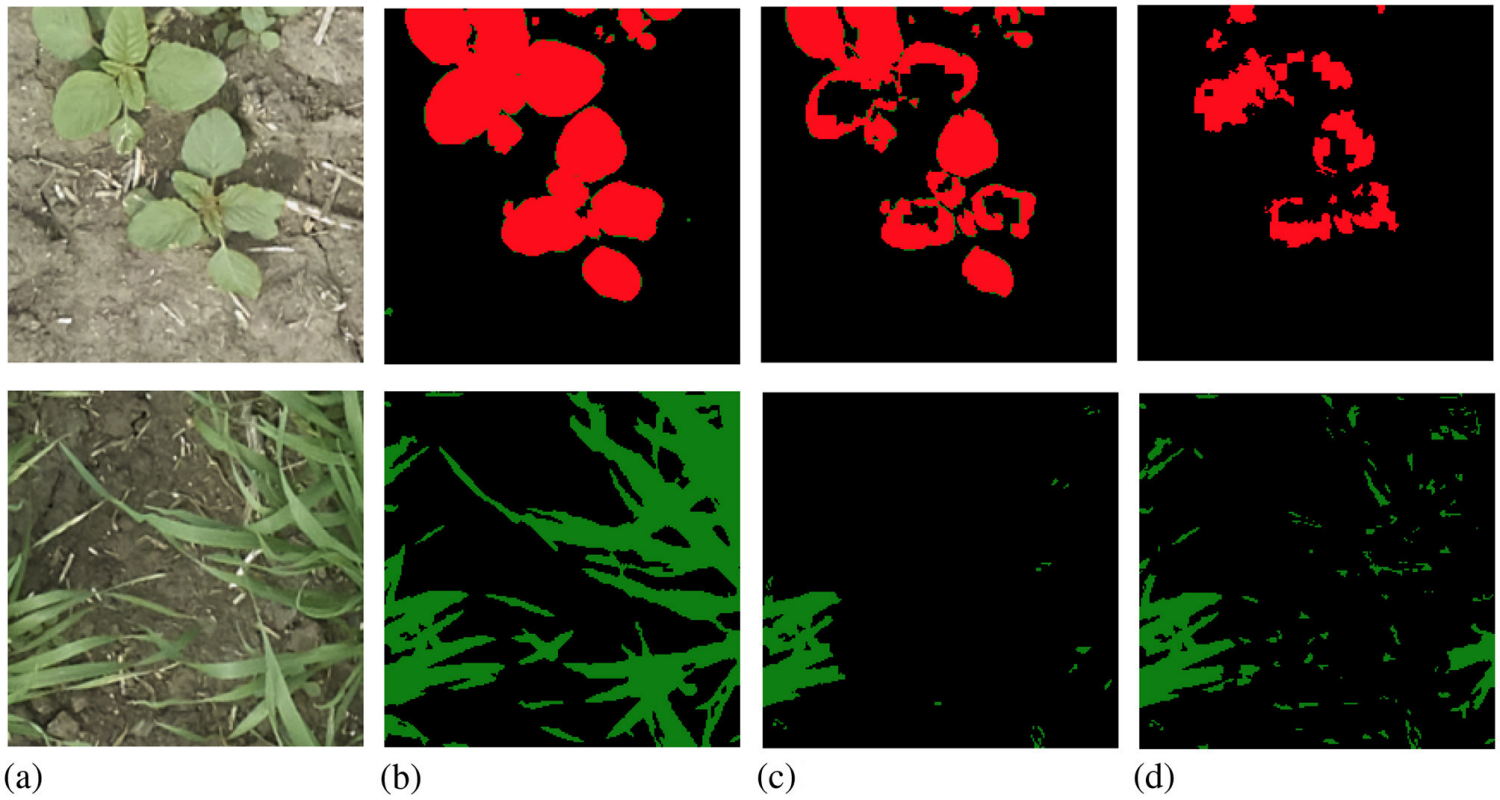
Examples from low-budget Nassar experiments comparing dynamic-budget AL labels and static-budget AL labels. The top row shows static-budget querying under-labeling while the bottom row shows static-budget querying over-labeling due to the fixed budget. **(A)** RGB image. **(B)** Manual label. **(C)** Dynamic-budget. **(D)** Static-budget.

**Figure 7:**
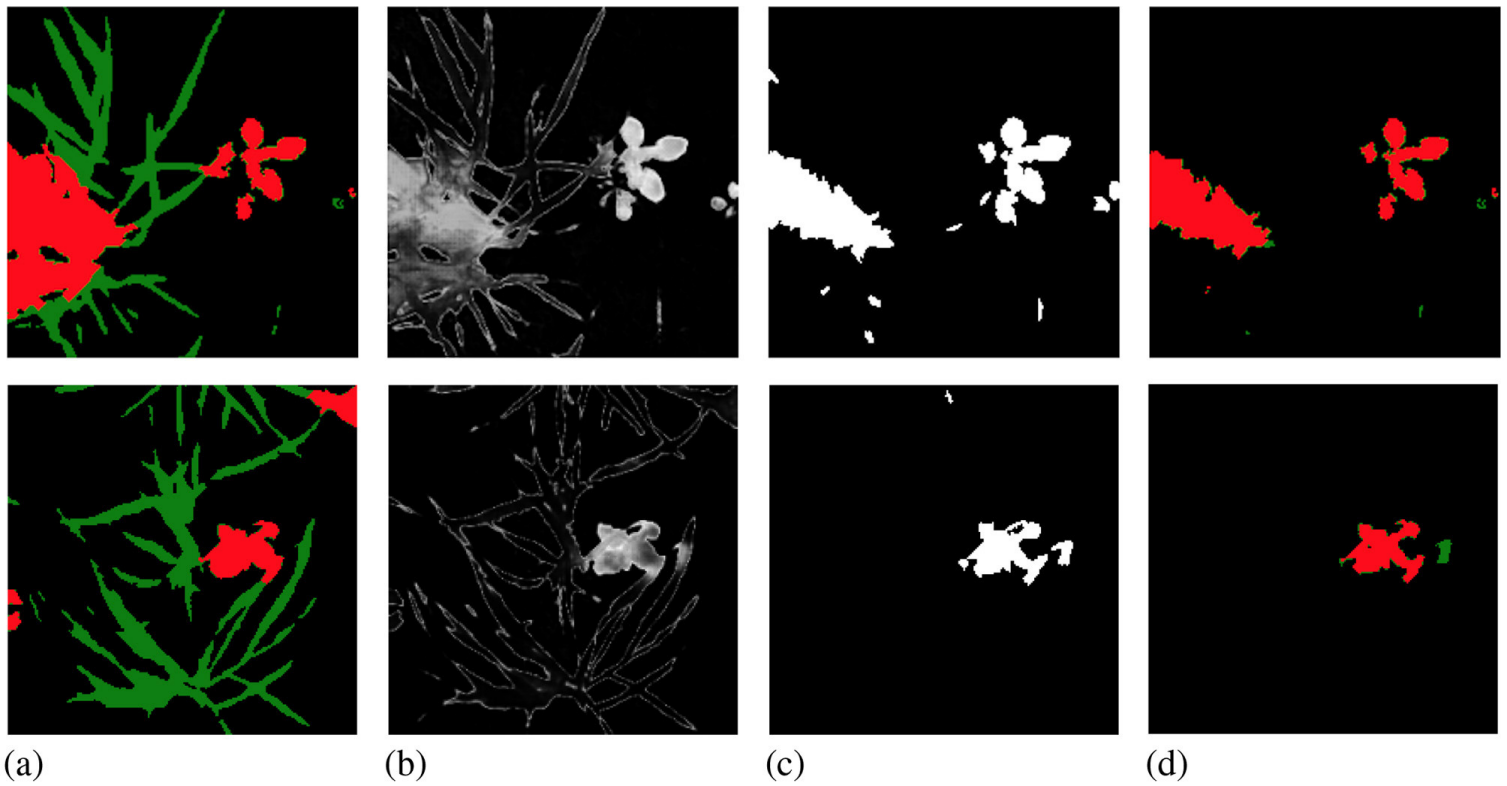
Examples from Nassar show foreground-only querying on whole-image manual label **(A)**. The per-pixel uncertainty map **(B)** and query mask **(C)** only include foreground pixels/superpixels. In the resulting active learning label **(D)** the model ignores most crop pixels. The lower part of the weed plant on the left of the top image and the two smaller weeds on the edge of the bottom image are also ignored.

**Figure 8:**
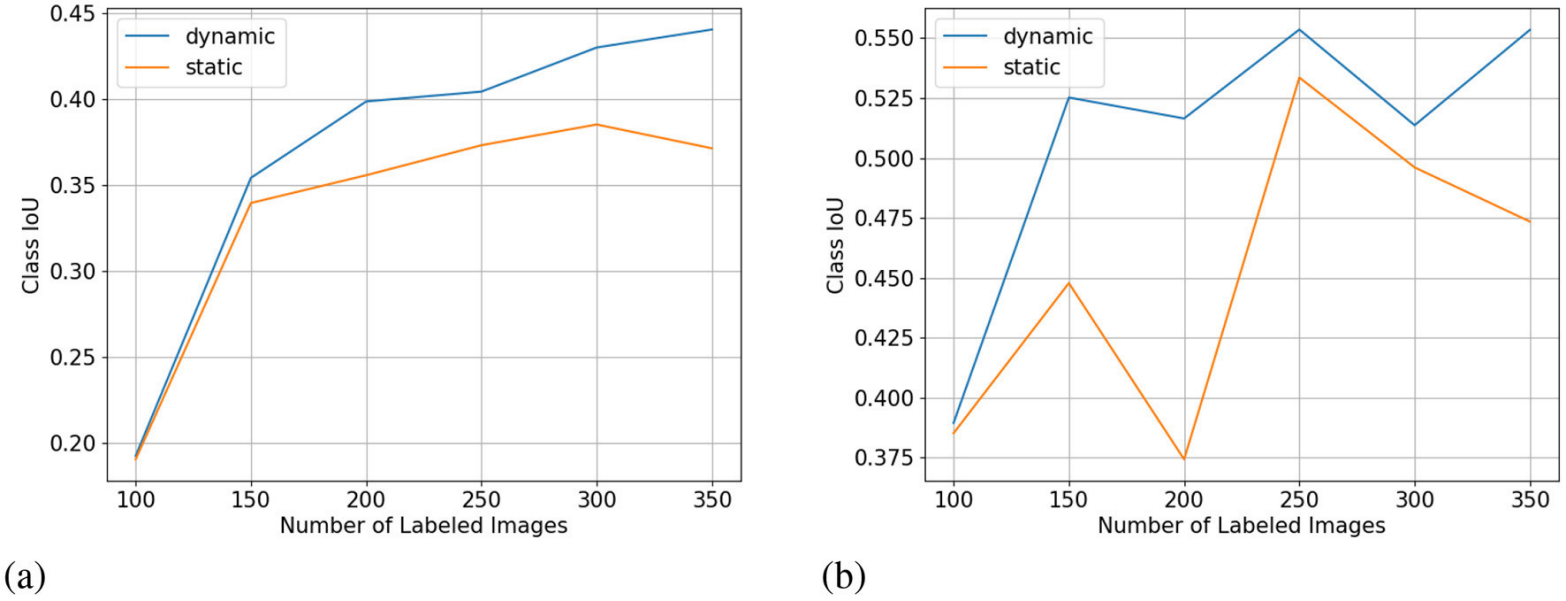
Fence **(A)** and truck **(B)** class IoU (higher is better) from the low-budget Cityscapes experiment. X-axis for all subplots is the number of labeled images.

**Figure 9:**
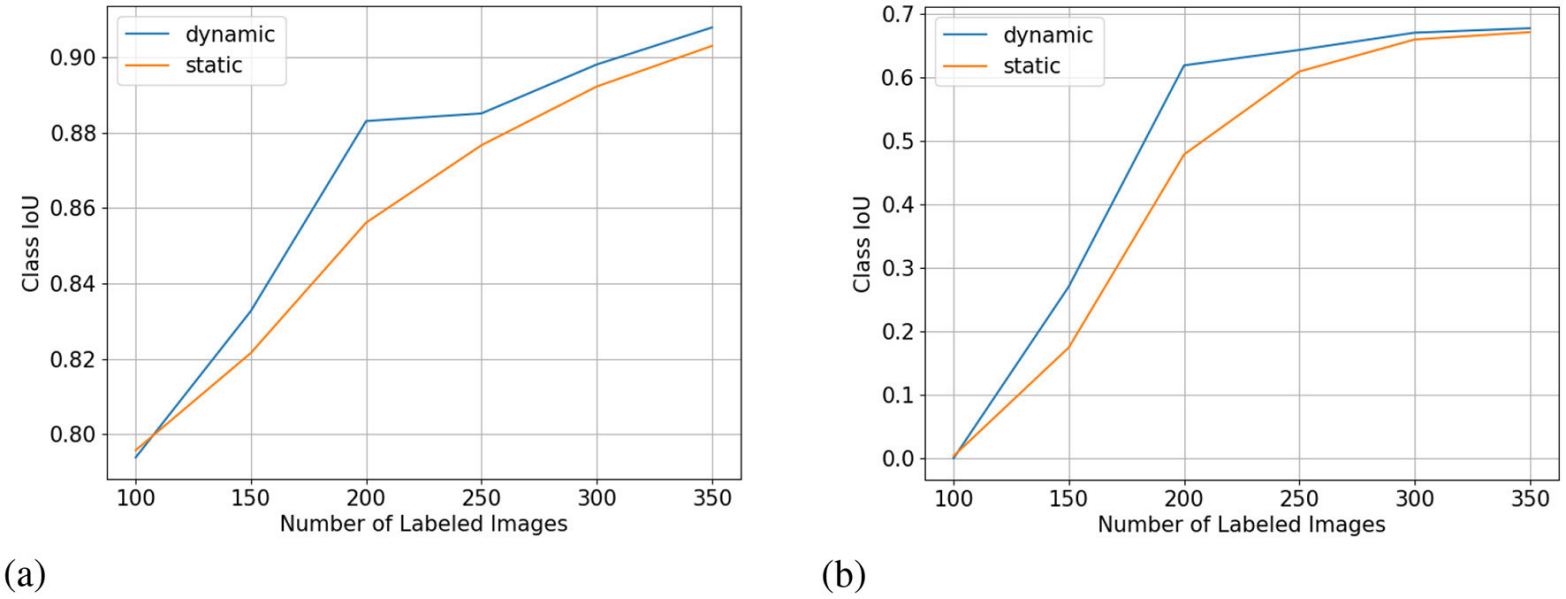
Crop **(A)** and weed **(B)** class IoU (higher is better) from the low-budget Nassar experiment. X-axis for all subplots is the number of labeled images.

We show the results of all low-budget experiments in graphs in Figure 5. The top graphs show the mIoU change with increasing labeling budgets of three experiments: dynamic-budget fine superpixels, static-budget fine superpixels, and dynamic-budget coarse superpixels. The red dashed line marks 95% of the 350 random whole-image manual label baseline. We chose the 95% baseline due to it being a common baseline used in AL experiments like Mackowiak et al. (2018). We show examples of our dynamic-budget querying strategy improving the over-/under-leveling problems found in static-budget querying in Figure 6. Figure 7 shows two examples of the foreground-only querying strategy described in Section 2.2.3. We also show fence and truck IoU curves from Cityscapes experiments in Figure 8 and crop and weed IoU curves from the Nassar experiments in Figure 9.

**Figure 10:**
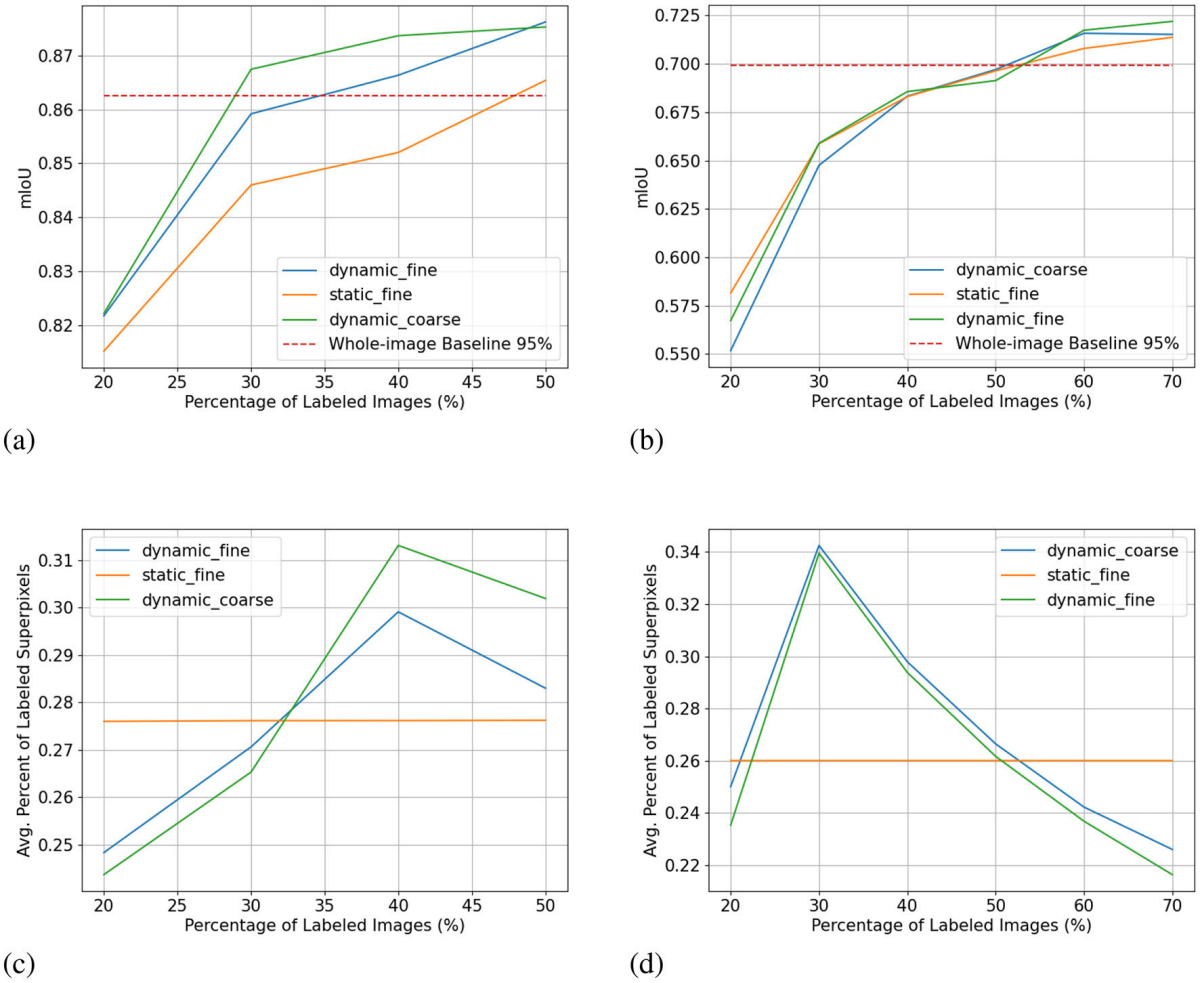
High-budget experiment results showing mIoU (top row, higher is better) and average percentage of labeled superpixels (bottom row) for the Nasser (left) and Cityscapes (right) dataset. Label dynamic_fine refers to dynamic-budget experiments using fine superpixels; label dynamic_coarse refers to dynamic-budget experiments using coase superpixels; label static_fine refers to experiments using fine superpixels. X-axis for all subplots shows the percentage instead of the number of labeled images to make the plots more readable. **(A)** mIoU on Nassar 2020. **(B)** mIoU on Cityscapes. **(C)** Avg. % Labeled Superpixles on Nassar 2020. **(D)** Avg. % Labeled Superpixles on Cityscapes.

The results of all high-budget experiments can be found in Figure 10. The graph layout follows that of the low-budget experiments. The red broken line for high-budget experiments marks 95% of a fully manually labeled whole dataset baseline.

### 3.1 Dynamic-budget querying

Dynamic-budget querying has a significant mIoU advantage over static-budget querying in low- and high-budget experiments on the Nassar dataset. The low-budget dynamic-budget experiment in Figure 5A shows an advantage of 3.6% mIoU at 150 labeled images and 5.6% mIoU at 200 labeled images over the low-budget static-budget querying experiment. The static-budget querying requires around 250 labeled images to reach 95% of the random 350 whole-image labels baseline which matches the accuracy of dynamic-budget querying at 200 labeled images. The low active learning step budget combined with the sparsity of foreground pixels in the Nassar dataset amplifies the over- or under-labeling problem with static-budget querying as shown in Figure 6. In this low-budget experiment, an average of 31% of foreground superpixels from the queried 300 images are labeled.

The difference between dynamic-budget querying and static-budget querying is smaller for the high-budget Nassar experiment shown in Figure 10A. Dynamic-budget querying maintains an advantage above 1% mIoU over static-budget querying after 30% of images are labeled. Dynamic-budget querying surpassed 95% of our fully labeled whole-dataset baseline with 30% of labeled data compared to static-budget querying requiring 50% of the images labeled. The high-budget experiment on average labels 28% of foreground superpixels from the queried 40% of 1,952 Nassar images.

The low-budget Cityscapes experiments in Figure 5B show dynamic-budget querying outperforms static-budget querying by 2.4% IoU at 100 and 150 labeled images. After 150 labeled images, the performance differences are negligible. Around 36% of superpixels in the queried 300 image are labeled in this experiment. The high-budget Cityscapes experiment (Figure 10B) did not show noticeable performance between dynamic- and static-querying. This could be a result of ample labeled data giving the algorithm a higher tolerance on less optimal labeling.

### 3.2 Coarse vs. fine superpixels

The mIoU difference between using fine superpixels and coarse superpixels is small. In the low-budget scenario shown in Figure 5C, coarse superpixels show a 2.7% higher IoU than fine superpixels at 150 labeled images on the Nassar dataset. However, the performance gain comes with a 2.3% higher foreground superpixel labeling percentage penalty. The two experiments show no notable performance difference for other active learning steps. The high-budget Nassar experiments in Figure 10C show < 1% IoU or superpixel labeling percentage difference at any active learning step.

The same can be observed from the Cityscapes experiments where high-budget experiments (Figure 10D) between coarse and fine superpixels have close or less than 1% IoU difference. Fine superpixels outperform coarse superpixels by 1.8% IoU in the low-budget testing (Figure 5D) at 300 labeled images. Both high- and low-budget Cityscapes experiments show that coarse superpixels have a higher superpixel labeling percentage, while the difference is insignificant.

## 4 Discussion

### 4.1 Low vs. high budget

Figure 5 shows our approach is more effective in a low-budget setting which, in our opinion is more important since reaching a reasonable accuracy with minimal labeling is the primary goal of AL. Especially in the low-budget Nassar experiments, we can reach a crop IoU of over 0.9 and a weed IoU of almost 0.7 with only 350 labeled images as shown in Figure 9. Our dynamic-budget querying scheme only needs 200 labeled images to surpass 0.6 weed IoU. These accuracies are often sufficient in many applications such as targeted spraying where the sprayer only needs to detect the locations of weed objects instead of needing perfect contours of these weed plants. Moreover, dynamic-budget and static-budget querying have identical computational complexity as the most expensive operation is the sorting of the superpixel uncertainty map. These low-budget experiments show that our dynamic-budget querying scheme allows the user to effectively train a model with minimal labeling effort and no additional computational cost.

Figure 10 shows our method is less effective in a high-budget scenario but high-budget AL queries can be less desirable due to diminishing performance gains. Even with a complex and challenging dataset like Cityscapes, we only need around half of the dataset to reach comparable accuracy to that of the fully labeled baseline. In fact, with only 350 labeled images, the low-budget dynamic-budget querying AL achieved a mIoU of 0.635 which is 86% of the fully labeled baseline with almost 3,000 images.

### 4.2 Effect on under-represented classes

One interesting observation of the per-class IoU is that under-represented classes in the Cityscapes dataset do not always have lower class IoU. Despite having extremely low pixel count and image appearance frequency, the bus class reached IoUs of 0.690 in the dynamic-budget experiment and 0.675 in the static-budget experiment at 350 labeled images. In comparison, the fence class with significantly higher pixel count and appearance only reached 0.440 in the dynamic-budget experiment and 0.371 in the static-budget experiment. We selected two classes to show the effect of dynamic-budget querying on individual classes. Figure 8A shows the IoU curve of the fence class which has a low pixel count but a medium appearance frequency. The dynamic-budget experiment maintains a visible advantage over the static-budget experiment after 150 labeled images, ending with 6.9% higher class IoU at 350 labeled images. Figure 8B shows the IoU curve of the truck class which is one of the least represented classes in the Cityscapes dataset. The dynamic-budget experiment converged after 150 labeled images and its class IoU stabilized around 0.52 to 0.55. The static-budget experiment showed greater instability where the class IoU showed two cases of significant IoU decrease at 200 labeled images and 350 labeled images despite an increase in labels. These two examples show that dynamic-budget querying not only can result in higher IoU but also more stable performance, especially for the less represented classes.

Figure 9 shows the IoU curve of the two foreground classes in the low-budget Nassar experiment. The per-class IoU change follows the trend of the mIoU curve where at 200 labeled images dynamic-budget querying shows the largest performance advantage over static-budget querying. The more dominant crop class showed a high class IoU of over 0.8 for both experiments, with dynamic-budget querying having a 2.7% IoU gain at 200 labeled images. The sparsely distributed weed class converged around a class IoU of 0.67. For the weed class, dynamic-budget querying shows a more noticeable 14% IoU advantage over static-budget querying.

### 4.3 Effect of superpixel sizes

Even though none of the experiments showed in Figures 5C, 10D exhibit clear performance or budget advantage, the real-world labeling cost of using coarse superpixels could be higher. Finer superpixels are more likely to contain pixels of the same class which can be labeled with one click, while coarse superpixels can contain multiple classes and require more steps to label. However, using coarse superpixels means having a lower superpixel count per image, resulting in fasting sorting of the superpixel uncertainty map in the querying stage.

### 4.4 Related works

Active learning can be broadly classified into stream-based sampling and pool-based sampling. Stream-based sampling evaluates each data point independently (Cheng et al., 2013) while pool-based sampling ranks the entire dataset before making a decision. By examining and comparing each data point in the dataset before making a decision, pool-based sampling can select only the most impactful data points with a trade-off of larger ranking overhead. Pool-based active learning is typically an iterative process with four key components: the unlabeled pool, the labeled AL training set, the oracle (human labeler) and the learner (deep learning model) (Settles, 2009). The learner uses querying strategies to rank and select a batch of the most beneficial samples from the unlabeled pool to be labeled by the oracle. Then these newly labeled samples are added to the AL training set to train the model. This process can be repeated until the model is trained to satisfactory accuracy or a preset annotation budget is exhausted.

The querying strategy which dictates the effectiveness of active learning algorithms can be split into two major categories: diversity sampling and uncertainty sampling. Diversity sampling methods are usually model agnostic and depend on extracted features from unlabelled images to diversify the training set (Sener and Savarese, 2017a). The uncertainty sampling algorithm used in our project uses a trained model to find the most uncertain samples for labeling. The effectiveness of an uncertainty sampling algorithm relies heavily on the quality of model uncertainty measured with acquisition functions. In this paper, we use Bayesian Active Learning by Disagreement (BALD) (Houlsby et al., 2011) as our acquisition function, which is a combination of the basic max entropy approach and sampling disagreement.

**Figure 11:**
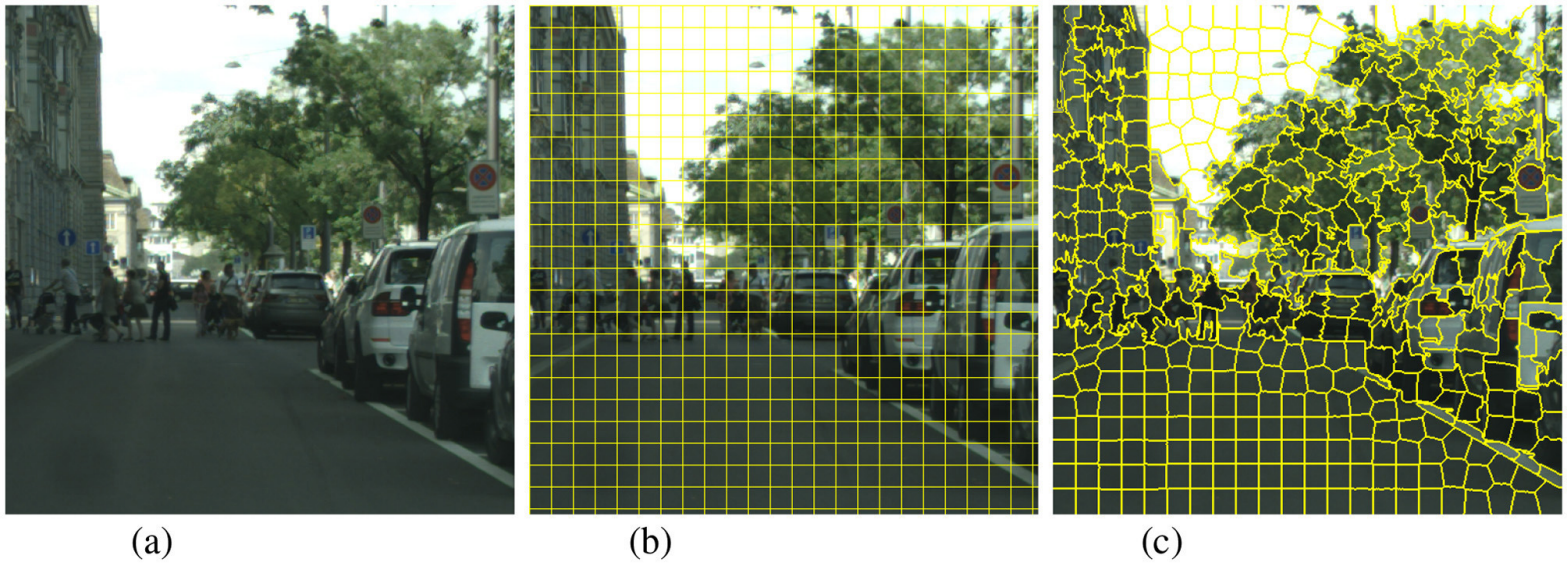
Different querying units are shown with a cropped sample from Cityscapes. Superpixels generally follow the contour of objects closely while rectangular grids regularly cross object contour boundaries. With large enough segment counts, most superpixels will only contain pixels from one single class while it is common for a rectangular grid to contain pixels from different classes. **(A)** Whole image. **(B)** Rectangular grid. **(C)** Superpixel (SLIC).

Most active learning algorithms, like the popular Cost-Effective Active Learning (CEAL) (Wang et al., 2016), are designed for image classification tasks and naturally query full images. Although they can be easily adapted to semantic segmentation tasks, we can further improve the active learning algorithms to query individual pixels, or more commonly rectangular regions or superpixels for efficiency as visualized in Figure 11. Cost-Effective REgion-based Active Learning (CEREALS) (Mackowiak et al., 2018) applies this idea to CEAL by querying rectangular grids in an unlabelled image. An alternative to querying rectangular grids is to query segmented regions called superpixels. Superpixels are generated by segmentation algorithms which split the image into clusters of pixels that are similar to each other. Some commonly used superpixel algorithms include Felzenszwalb's method (Felzenszwalb and Huttenlocher, 2004), SLIC (Achanta et al., 2012), and Compact Watershed (Neubert and Protzel, 2014).

### 4.5 Limitations and future works

One of the limitations of this work lies in our budget calculation where we use the number/percentage of superpixels/images as the labeling cost. This is an approximation of the real-world labeling cost as different superpixels can require different levels of labeling efforts. Instead of using the provided labels to simulate the manual labeling process, we plan to conduct future experiments with manual labeling to provide a more accurate labeling cost measurement.

In this paper, we used an agriculture dataset and a street scene dataset to show the generalizability of our proposed querying strategy. Different agricultural datasets can vary significantly in vegetation coverage, density, color and shape. These dataset-specific traits could influence the accuracy of our trained model, resulting in variations in the uncertainty estimation quality. We plan to test this querying strategy on other agriculture datasets with various plant species and background conditions in the future.

Our proposed querying strategy is versatile and can be easily applied to other AL algorithms. MC-Dropout along with other ensemble-based uncertainty estimation methods are also flexible and generally model agnostic where they can be used on more sophisticated models like visual transformers (ViTs). This makes testing our querying strategy with different combinations of uncertainty estimation methods and models quite straightforward as the common last step is to rank the resulting pixel-wise uncertainty maps. We plan to test the generalizability of our querying strategy with other models, uncertainty estimation methods and acquisition functions.

Active learning is highly data-efficient when only labeling costs are considered. However, active learning algorithms can also be wasteful since most of the already collected images in our unlabelled pool are ignored. Another promising future direction is to combine our active learning approach with self-supervised learning (SSL) to utilize all the unlabelled data. We could use the entire unlabelled dataset to perform SSL pre-training to initialize model weights and then take advantage of active learning to construct a compact and informative dataset for the fully-supervised downstream task. By complementing active learning with self-supervised learning, we could make use of all the collected data and potentially maximize data efficiency. Moreover, the self-supervised pre-training process could be a superior alternative to the random initialization for active learning. Since active learning is an iterative process, a poorly initialized model could negatively affect the active learning process. Creating a more robust initialization state with self-supervised learning could further enhance the effectiveness of active learning algorithms.

### 4.6 Conclusion

In this paper, we introduced a novel dynamic-budget superpixel querying strategy for regional active learning algorithms. We showed that dynamic-budget querying can be effective compared to fix-budget querying in a low-budget scenario or on foreground-pixel sparse datasets like agricultural field images (Nassar 2020). Our low-budget experiments show a maximum of 5.6% (Nassar) and 2.4% (Cityscapes) IoU improvement when switching from static-budget querying to dynamic-budget querying. This advantage diminishes when the algorithm is given a higher budget with more labeled data. We did not find significant performance or labeling cost differences between using coarse and fine superpixels. This simple yet effective dynamic-budget querying strategy can be easily adapted to other regional active learning algorithms to improve the querying efficiency.

## Data Availability

Publicly available datasets were analyzed in this study. This data can be found here: the Nassar 2020 dataset can be found at https://www.kaggle.com/datasets/yueminwang/nassar-2020. The Cityscapes dataset can be found at https://github.com/mcordts/cityscapesScripts.
